# Metasurface wavefront control for high-performance user-natural augmented reality waveguide glasses

**DOI:** 10.1038/s41598-022-09680-1

**Published:** 2022-04-06

**Authors:** Hyunpil Boo, Yoo Seung Lee, Hangbo Yang, Brian Matthews, Tom G. Lee, Chee Wei Wong

**Affiliations:** 1 Mesoscopic Optics and Quantum Electronics Laboratory, University of California, Los Angeles, CA USA; 2 Nanofabrication Laboratory, University of California, Los Angeles, CA USA

**Keywords:** Displays, Optoelectronic devices and components

## Abstract

Augmented reality (AR) devices, as smart glasses, enable users to see both the real world and virtual images simultaneously, contributing to an immersive experience in interactions and visualization. Recently, to reduce the size and weight of smart glasses, waveguides incorporating holographic optical elements in the form of advanced grating structures have been utilized to provide light-weight solutions instead of bulky helmet-type headsets. However current waveguide displays often have limited display resolution, efficiency and field-of-view, with complex multi-step fabrication processes of lower yield. In addition, current AR displays often have vergence-accommodation conflict in the augmented and virtual images, resulting in focusing-visual fatigue and eye strain. Here we report metasurface optical elements designed and experimentally implemented as a platform solution to overcome these limitations. Through careful dispersion control in the excited propagation and diffraction modes, we design and implement our high-resolution full-color prototype, via the combination of analytical–numerical simulations, nanofabrication and device measurements. With the metasurface control of the light propagation, our prototype device achieves a 1080-pixel resolution, a field-of-view more than 40°, an overall input–output efficiency more than 1%, and addresses the vergence-accommodation conflict through our focal-free implementation. Furthermore, our AR waveguide is achieved in a single metasurface-waveguide layer, aiding the scalability and process yield control.

## Introduction

Augmented reality (AR) and mixed/merged reality (MR) devices enable the user to see both the real world and virtual images simultaneously, leading to considerable interest as next-generation mobile wearable devices beyond the smartphone^1–6^. Benchmarked by the iconic Google Glass, there have been many multifunctional displays including recent efforts by Microsoft Hololens and Magic Leap. A key element in the smart glasses is the optical waveguide that delivers virtual images from the display source to the eye, augmenting our vision through the transparent optical glasses. For compactness, recent optical elements have utilized holographic and diffractive optical elements (HOEs and DOEs) instead of bulky free-space optics headsets such as birdbath designs using bulk lenses and mirrors. These HOEs and DOEs have recently been implemented in various diffraction grating structures such as Bragg, volume, surface relief and blazed gratings^7–28^.

AR devices with HOEs and DOEs serve as next-generation display technology, with improving display resolution, lower time-delay in images, and cost scalability^29,30^. While the AR display and sensing efforts benefited from the smart phone technology developments, most HOE and DOE-based AR devices still have physical performance limitations—such as insufficient display resolution, small field-of-view, low input–output efficiencies, and manufacturing yield-scalability^31,32^—hindering their wide-spread adoption. Furthermore, the vergence-accommodation conflict (VAC) is a common and important challenge for head-mount displays (HMDs)^33–37^. Our eyes use multiple depth cues to conceive the depth of an image, consisting of an accommodation depth (wherein our eyes adjust the lenses foci to acquire a clear image) and a vergence depth (based on the angle between our eyes to perceive a distance). For real-world images, the accommodation and vergence distances are the same. For AR display images, however, the virtual image comes from the display plane and not at the depth of the image. Hence, the binocular disparity to setup the image’s virtual focal distance (vergence distance) does not match the virtual image source’s actual position (accommodation distance). In trying to match the accommodation power to the vergence distance, this results in blurred images, fatigue and nausea in our AR-MR imaging perception, especially for long-term display usage. Maxwellian-view displays, by projecting transmissive displays directly on the retina, can alleviate this conflict by reducing the dependence on the eye lens accommodation^38–41^ but are often bulky free-space optical elements and with small field-of-view.

Metasurfaces can overcome the physical limitations of HOEs and DOEs, including our implementation of focal-free metalenses to overcome the vergence-accommodation conflict while preserving a large field-of-view. Conventional HOEs and DOEs utilize periodic grating structures for multiple beam diffraction, fine-tuned for red-blue-green wavelengths simultaneously. To achieve multiple focal positions, multiple HOE/DOE waveguides are implemented simultaneously, a complexity that can result in lower display quality from unwanted diffractions and multi-step fabrication processing. Metasurfaces, with spatially-configured arrays of subwavelength-scale optical scatters, are an alternative platform for direct wavefront shaping^42–50^. The unique versatility and multi-functionality of the metasurface platform have thus led to advances in flat-optics devices, all-dielectric platforms, polarization-spatial conversion, quantum photonics, metalenses^51–67^, and AR visors^68–70^. Utilizing the building blocks within the metasurface array, metasurface geometrical parameters—such as the size, shape and orientation—can control the reflected and transmitted wavefront from first-principles^71–94^. The subwavelength orientation also reduces the generation of spurious diffraction orders versus those observed in HOEs and DOEs, resulting in a higher waveguide input–output efficiency and suppression of unwanted effects such as virtual focal points, halos, and ghost images^95–107^.

Here we describe our metasurface waveguide for high resolution, field-of-view (FoV), and output efficiency, while preserving a focal-free operation. Our metasurface optical elements (MOEs) are implemented for full-color operation, with the careful dispersion control in the excited propagation and diffraction modes, through analytical–numerical modeling, rigorous nanofabrication, and measurement. Each pixel in the input and output MOEs is carefully optimized for the collective high resolution and output efficiencies, while realizing a focal-free Maxwellian-view display system to overcome the vergence-accommodation conflict. Implemented in a CMOS-compatible cleanroom and foundry service, our MOE waveguide simultaneously preserves a large FoV across the full-color gamut, in our multi-period single-layer demonstration.

## Results

**Figure 1:**
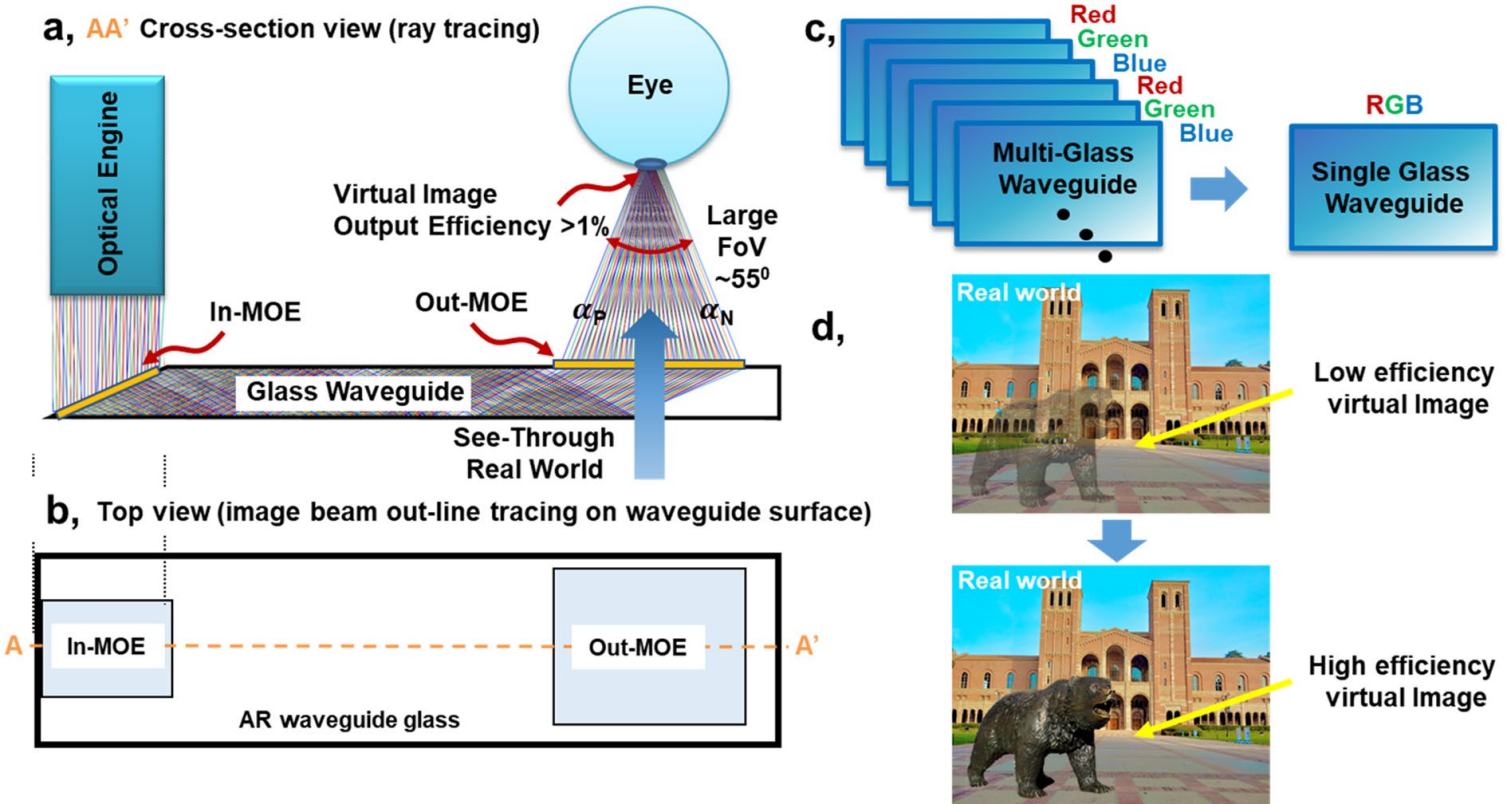
(**a**, **b**) Overview of our metasurface AR/MR waveguide glass architecture with cross-section and top views of the ray propagation, respectively with the cross-section area shown as AA′ in the top view. (**c**) Single glass waveguide compared with conventional multi-glass waveguide for each color and each focal plane. (**d**) Efficiency comparison for virtual image.

Figure 1 shows the key architecture and approach of our MOE AR display waveguide, with the AA’ cross-section (Fig. 1a) and top-view (Fig. 1b) highlights in the display beam propagation. First, we note that, by incorporating careful design of each pixel for the MOEs, the RGB spectrum is guided through the eye lens to the retina, enabling a close to Maxwellian-view operation, for focal-free display to address the vergence-accommodation conflict. Second, our designed MOE display has an FoV determined only by our metasurface grating structure, achieving ≈ 55° currently or higher, even with normal-index glass. Third, as shown in Fig. 1a, the In-MOE is at a slant angle on the glass waveguide to direct the wave propagation towards the Out-MOE. Without the slant angle, the diffraction would be in both positive and negative directions (of the MOE surface normal), leading to a lower (approximately half) efficiency. Together with rigorous coupled-wave analysis to minimize undesired diffraction modes, we are able to maximize the efficiencies of the desired modes towards the output, surpassing efficiencies more than 1%. This aids the embedding of augmented information and virtual images with real-world images, especially with outdoor light, as shown in Fig. 1d example. We also note that prior HOE-DOE AR waveguides, with multi-layer multi-glass waveguides, have spurious diffraction modes and hence efficiencies sizably lower than 1%, necessitating indoor operation or shielding outdoor light by 80%. Fourth, as shown in Fig. 1a, c, our MOE waveguide utilizes only a single glass layer for the whole RGB spectrum, reducing unwanted diffraction and with higher efficiency. Our single-layer implementation also brings compactness and lightweight operation, while simplifying the MOE fabrication and yield.

**Figure 2:**
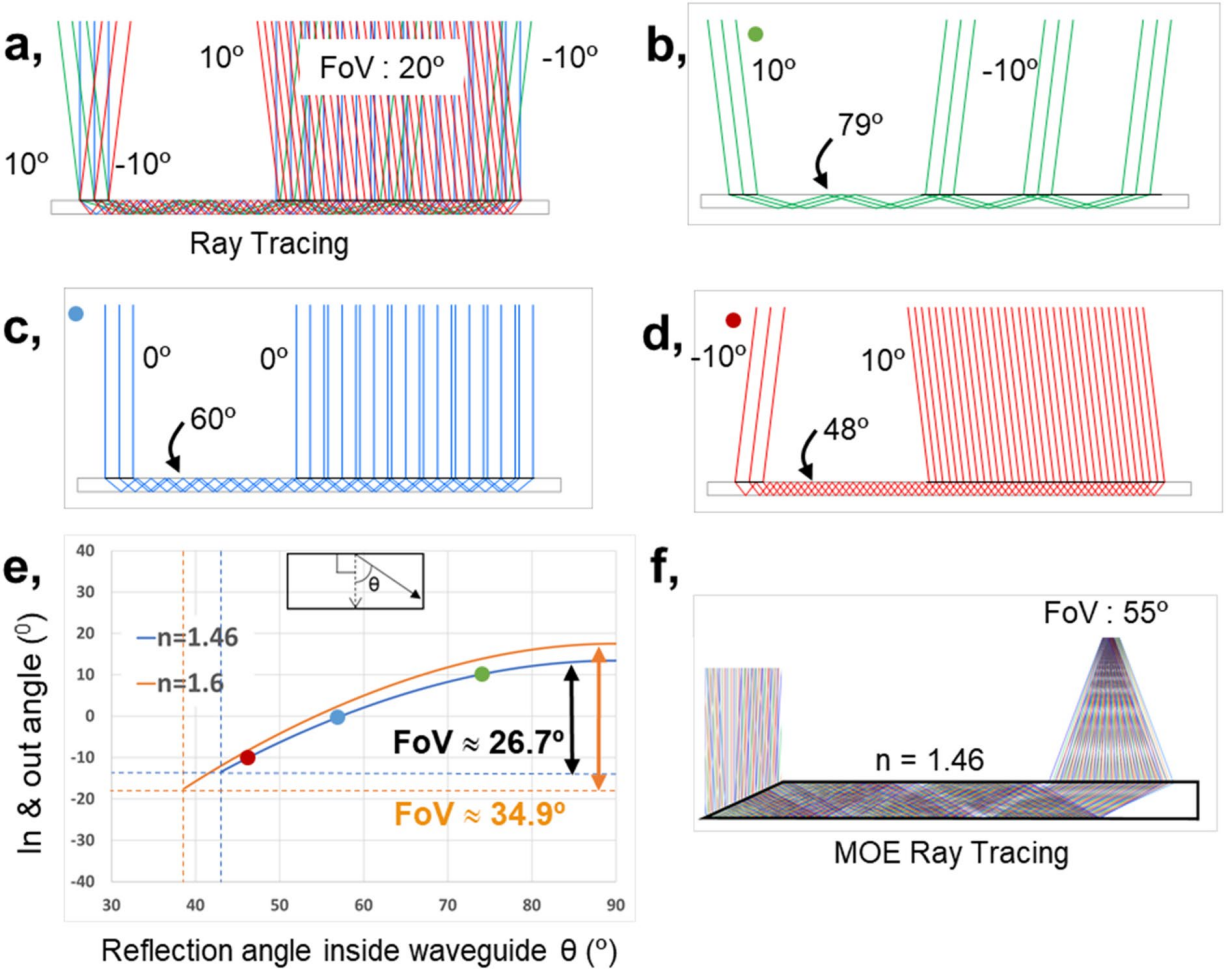
Reconstructed total internal reflection and input–output propagation of the conventional optical element waveguide. The chief ray angle is illustrated. (**a**) Overall input–output ray propagation. (**b**–**d**) Comparisons of 10°, 0°, and − 10° propagation (**e**) Designed FoVs for different waveguide glass refractive indices, versus angle in waveguide. The dashed vertical lines show the total internal reflection bounds for the two corresponding refractive indices. (**f**) Ray tracing of MOE for 646 nm showing FoV of 55°.

We first start by defining the FoV bounds from conventional total internal reflection of a waveguide^25,108–110^, without a metasurface. As illustrated in Fig. 2a–d, for a ± 10° input, the ray-traced output would be $\mp$ 10°; one can obtain an increased output FoV with larger input angles. However, the waveguide total internal reflection requirement bounds the input positive angles to less than 13.3°. This limits the FoV to less than or ≈ 26.7°, for a glass waveguide of 1.46 refractive index. For one of the higher-index glasses at 1.6 refractive index as shown in Fig. 2e, the FoV can be increased to 34.9° but rapidly faces an asymptotic limit. By controlling the wave propagation through multi-period metasurfaces, Fig. 2f shows the increased of the FoV to ≈ 55° for a normal-index glass at 1.46 refractive index. This illustration is for 646 nm, supplementing the 520 nm overview shown in Fig. 1a. Through rigorous coupled-wave analysis (RCWA)^111–114^, simple modal method (SMM)^115–120^, and finite-difference time-domain (FDTD)^121^, we optimize our MOE at each position for each wavelength. With the distance from the out-MOE to the eyebox set by design and a desired FoV fixed, we fine-tune our MOE grating periods for the desired input–output angle at each pixel, for each wavelength. We note that our MOE implementation is mostly bounded by nanofabrication lithography resolution.

**Figure 3:**
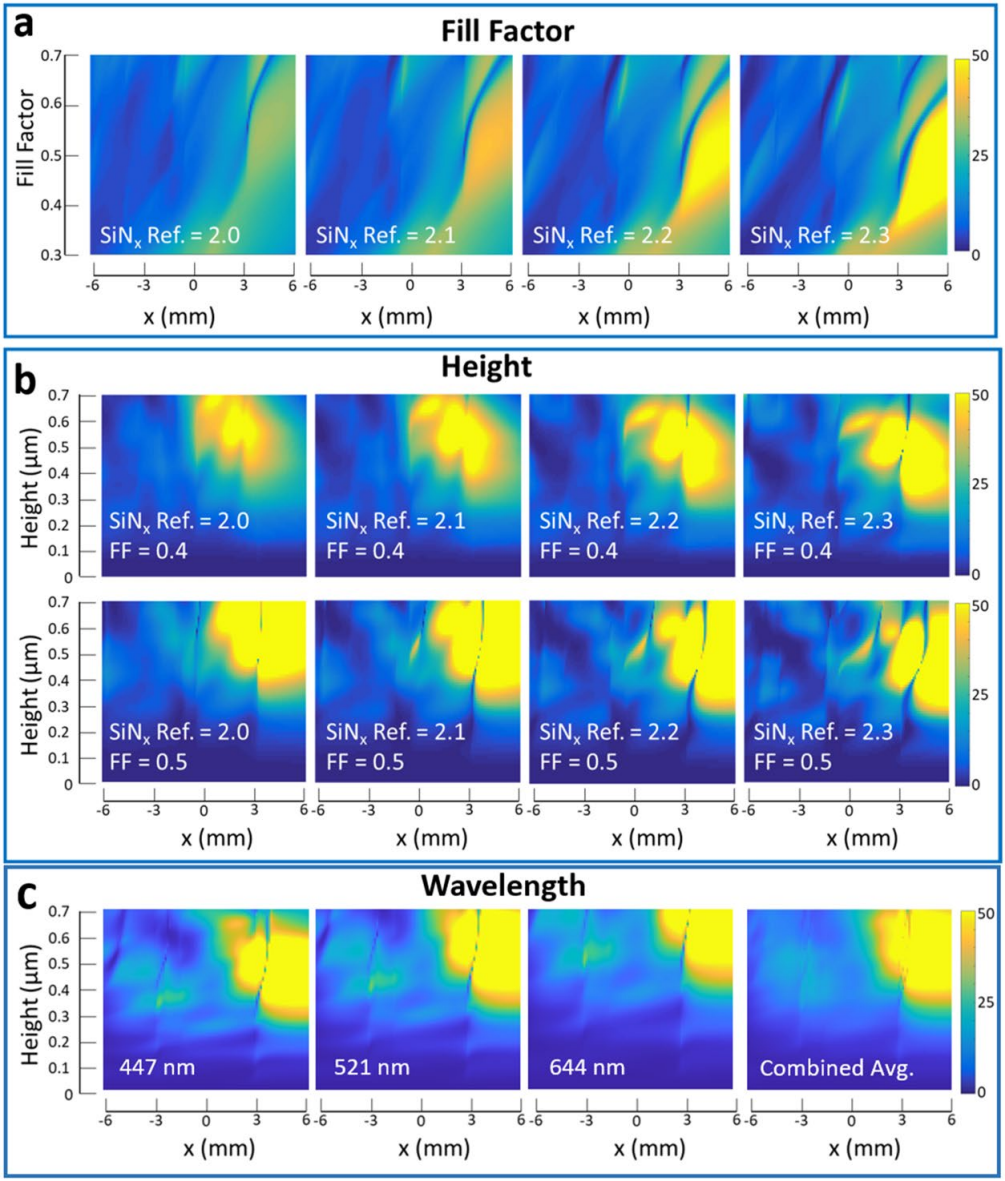
Numerical design map optimization of the metasurface waveguide glass, showing the metasurface optical waveguide display diffraction efficiencies for input/output MOE in the waveguide propagation direction for different positions in the waveguide *x*, fill factor (FF), and height parameters. Rigorous coupled-wave analysis (RCWA) numerical simulation is used in each design point of the 2D maps. (**a**) The height is fixed at 0.35 nm. Color scale bar denotes total intensity input–output propagation efficiencies in percentage. (**b**) Top panel for a fill factor (FF) of 0.4 and bottom panel for a fill factor of 0.5, with varying waveguide heights. The same color scale bar is used. In this implementation, optimal regions for the waveguide heights, fill factors and refractive indices of the metasurface are denoted. (**c**) Fill factor 0.46 and SiN_x_ refractive index of 2.14 (at wavelength 521 nm) with wavelengths of 447 nm, 521 nm, and 644 nm for blue, green, and red, respectively and the combined average.

Figure 3 next shows the subsequent MOE efficiency computation and optimization for different propagation *x*-positions along the metasurface *y*-center, mapped for a range of SiN_x_ refractive indices, fill factors and SiN_x_ film heights. Our MOE incorporates a pixel-by-pixel phase control by introducing phase changes within a length of the wavelength. The abrupt phase shifts enable freedom in controlling the wavefront, with the propagation of light being governed by Fermat’s principle^63^. We observe that the efficiency is higher on the right side (positive-*x*) of each MOE and go up to even 50% total input–output efficiencies. This can be explained in Fig. 1a, where the input–output beams on the out-MOE is shown, for our slanted input MOE design. In a slanted input and via the Littrow mounting effect, the output positive–negative diffraction lobes have unequal efficiencies, with the *α*_P_ (positive diffraction lobe) having lower diffraction efficiencies than the *α*_N_ (negative diffraction lobe, more than the surface normal and 90°, in the reverted beam direction). These 2D maps are for a center wavelength of 520 nm and the *y*-center; other wavelengths and *y*-positions simply have an offset in the efficiency map or with perturbed efficiencies.

In optimizing for the high-quality images for AR devices, we observe two features in these 2D map plots—the average efficiency and the dark low-efficiency crossing lines. The efficiency fluctuations, including the dark crossing lines, can be understood when comparing the grating to the simple case of a slab waveguide, where a wave excites discrete modes in the gratings^113^. The diffraction properties and efficiency are mainly determined by the modes within the grating region. These modes propagate through the grating region with different effective indices and couple out at the grating-substrate interface^108^. The behavior of these modes results in the fluctuations and also cause the sharp dips in the efficiency maps of Fig. 3. To choose an implementation for a desired overall design (FoV, full color, and glass refractive index prototype), parameters which result in high average efficiencies and lowest number of dark crossing lines must be accounted for. Higher refractive indexes have generally higher average efficiency trends, but the number of dark lines almost doubles when the refractive index increases from 2.0 to 2.3. The fill factor and height shift the position of the dark crossing lines and have optimal conditions for highest average efficiencies—represented as bright islands, where the brightest pixels are located. The change in optimal height of the MOE based on the wavelength (color) and the combined average efficiency map is shown in Fig. 3c. Similar trends in the map and an increase in optimal height with wavelength can be observed. Based on these modelled mapping results, the SiN_*x*_ refractive index of 2.14, a MOE height of 0.35 μm and a fill factor of 0.46 are chosen as the best conditions, avoiding most of the dark crossing lines in our display implementation. With each of the input/output MOE having a maximum simulated efficiency close to 50% and a diffraction efficiency division between the three colors (one third efficiency at each input/output), the theoretical efficiency is calculated to be close to 2.8%.

**Figure 4:**
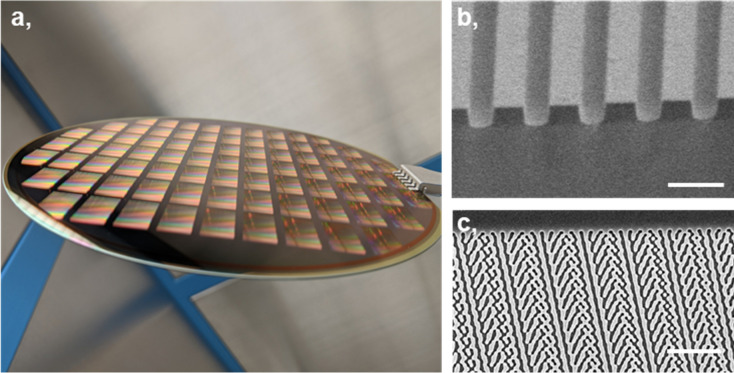
Nanofabricated metasurface wafer and optical elements. (**a**) Wafer-scale nanofabrication of the metasurfaces. (**b**) Cross-section scanning electron micrograph (SEM) of the engineered grating ridges on the glass wafer. Scale bar: 1 μm. (**c**) Top-view SEM of the optimized full-color metasurface elements. Scale bar: 5 μm.

Incorporating our AR waveguide design implementations, we fabricated the nanostructured metasurface optical elements in a silicon foundry process, with nitride film deposition, deep-ultraviolet lithography and MOE nanopatterning. Figure 4 summarizes our fabricated MOE wafer, with Fig. 4b illustrating the cross-section scanning electron micrograph (SEM) of our grating structures, and Fig. 4c illustrating the top-view zoom-in (SEM) of our optimized full-color MOEs. We note that the fabrication dimensions and specifications meet our designs. After each step in the fabrication, the MOE feature critical dimensions are carefully examined to confirm the design fidelity of each metasurface region to achieve the AR waveguide.

**Figure 5:**
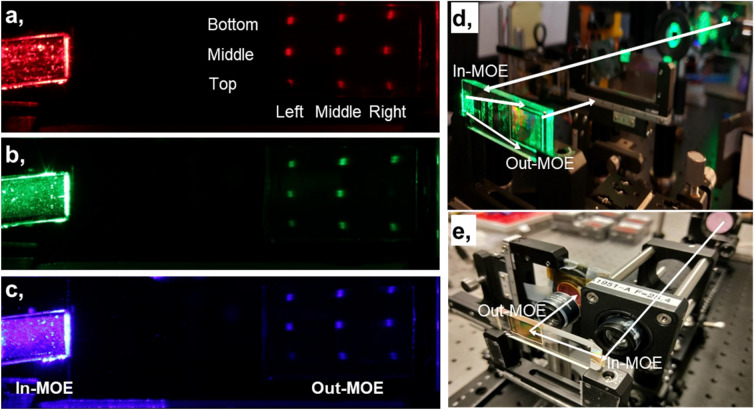
Measurement setups of the metasurface waveguide field-of-view, input–output efficiency, and modulation transfer function. (**a**–**c**) Calibration of the *f*_*x*_ and *f*_*y*_ focal lengths and in-coupling distortion, across the red–green–blue spectrum. Note, for panel (**c**) the blue laser input shows up as violet in this figure because of the color camera capture. (**d**) Setup for the field-of-view and input–output efficiency characterization. (**e**) Point source setup for the modulation transfer function characterization.

Figure 5 shows several experimental setups built specifically for the AR waveguide display characterization. First, Fig. 5a–c are the distortion calibration of the In-MOE and the focal length calibration of the Out-MOE, across the red–green–blue spectrum. The distortion calibration^122,123^ of the In-MOE is achieved by measuring the relative distances among nine laser spots at the Out-MOE. The small In-MOE distortion has little effect on the mono-MOE and the color-MOE. Based on the calibrated distortion, we modified the MOE and reduced the small distortions to the negligible levels by promoting the distortion calibration setup and optimizing the analysis software for the distortion calibration. The focal length calibration is achieved by tracking the nine laser spots out of the Out-MOE, which determines the focal spot astigmatism of the MOE, making it a key parameter for the design of our MOE. Figure 5d is the instrumentation for FoV^124–127^ and input–output efficiency characterization. After guiding the collimated laser beam into the In-MOE, we can obtain the FoV by scanning the beam size near the focal plane of the Out-MOE. The input–output efficiency is obtained by measuring the input–output power. Via changing the input beam size and position on the In-MOE, we can obtain the whole efficiency map of the MOE, which helps to improve the uniformity of the MOE. Figure 5e shows the setup we built for the modulation transfer function (MTF) characterization based on a point source mapping^123^. A focused laser beam passes through the In-MOE, the Out-MOE and an eye-equivalent lens, with imaging on a CMOS camera.

**Figure 6:**
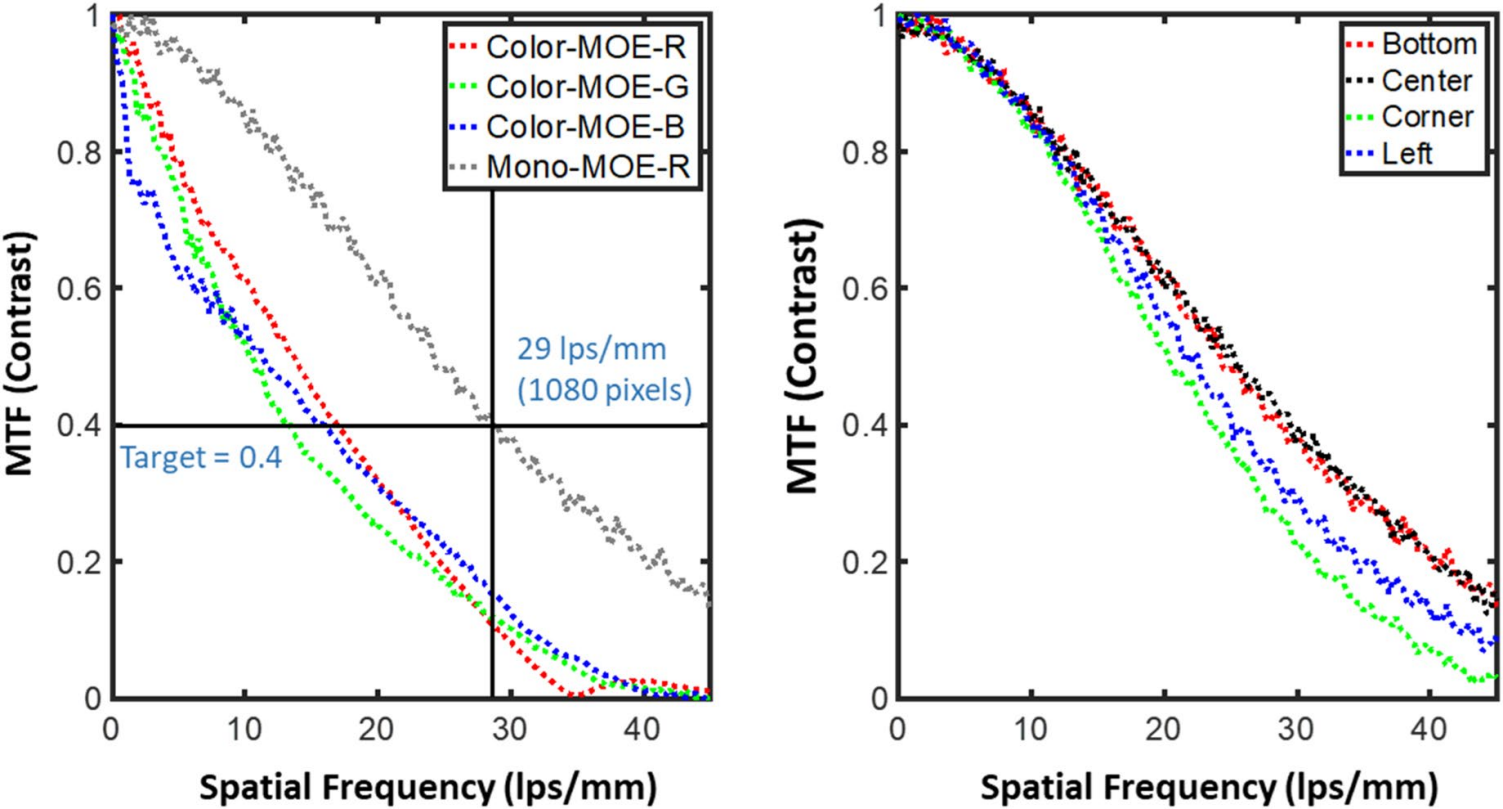
Measured modulation transfer function of metasurface waveguide prototypes: (**a**) Mono-MOE (red spectrum) and color-MOE (across the red–green–blue spectrum). (**b**) Mono-MOE (red spectrum) is compared across four positions in the MOE (MOE is symmetric with mirror plane at center in y direction). The target contrast is 0.4 at 27 lps/mm which is the full high-definition 1080-pixels for displays. For a contrast at 0.4, a 1064 × 1064 pixel display (≈ 29.0 lps/mm) for the red mono-MOE and a 520 × 520 pixel display (≈ 13.0 lps/mm, mainly limited by green) for the color-MOE are experimentally observed.

By Fourier analysis of the measured images, Fig. 6 shows the quantified MTF of our MOE waveguide. MTF specifies how the relative contrast of different spatial frequencies is handled by the system of our MOE display^6^. Here we select a Sony laser projector (MP-CL1, update rate per image of 60 Hz, resolution of 1920 × 720, and projects 43,200 lines/s) for the compact optical engine. In this setup we remove limitations from the optical engine on the MTF caused by limited capability to balance all aberrations using off-the-shelf lenses. These aberrations include spherical aberration, chromatic aberration, astigmatism, field curvature, and distortion. The contrast sensitivity of the human eye depends on several conditions such as the luminance, the viewing angle of the object, and the surrounding illumination^128–130^. Based on our model, we aggregate these conditions and assume the contrast sensitivity of the human eye is above 0.4^131^ when the resolution of image is below 1080 pixels (full resolution). In addition, the average diameter of human retina is 24 mm^132^ and its effective diameter within the FoV of our MOE is 20 mm. Therefore, we set the MOE display contrast target at 0.4, with a desired spatial frequency up to 27 lps/mm [1080pixels ÷ 2(pixels/lps) ÷ 20 mm].

Illuminated by a focused red laser, the measured MTF of the mono-MOE is depicted as a grey plot in Fig. 6a. For a contrast at 0.4, a 1064 × 1064 pixel display (≈ 29.0 lps/mm) of the red mono-MOE is experimentally observed. It is closely above our target resolution 1080 × 1080. For a contrast at 0.4 and our color-MOE, the display shows an experimental 520 × 520 pixel resolution. This is from the ≈ 13.0 lps/mm, mainly bounded by the green segments of our MOE; the red and blue segments are higher resolution at ≈ 17.0 and ≈ 15.3 lps/mm respectively in this proof-of-principle demonstration. This green-segment resolution of the color-MOE can be improved via optimization of fabrication to increase input–output efficiency. The complex color-MOE fabrication has a lower resolution currently compared to the mono-MOE because of beam overlap between the red–green–blue segments and the resulting reduced intensity contrast. In Fig. 6b, we show the comparison of the red mono-MOE across four different positions; center, left, bottom and corner. For a contrast at 0.4, the red mono-MOE shows the highest at the center and bottom of ≈ 29.0 and ≈ 30.4 lps/mm, respectively. The left edge and corner show slight degradation of resolution with ≈ 25.5 and ≈ 23.9 lps/mm, respectively, due to less input/output efficiency at those points. Consequently, the resolution of the edge and corner can also be increased similarly to the color-MOE. We also note that although the MTF of our system shown here is free of aberrations from lenses, the actual optical engine setup consisting of off-the-shelf lenses is currently mainly bounded by the slight mismatch between our optical engine and MOE caused by unbalanced aberrations. To overcome this limitation, later we will use customized lenses to optimize the optical engine to our MOE.

## Discussion

**Figure 7:**
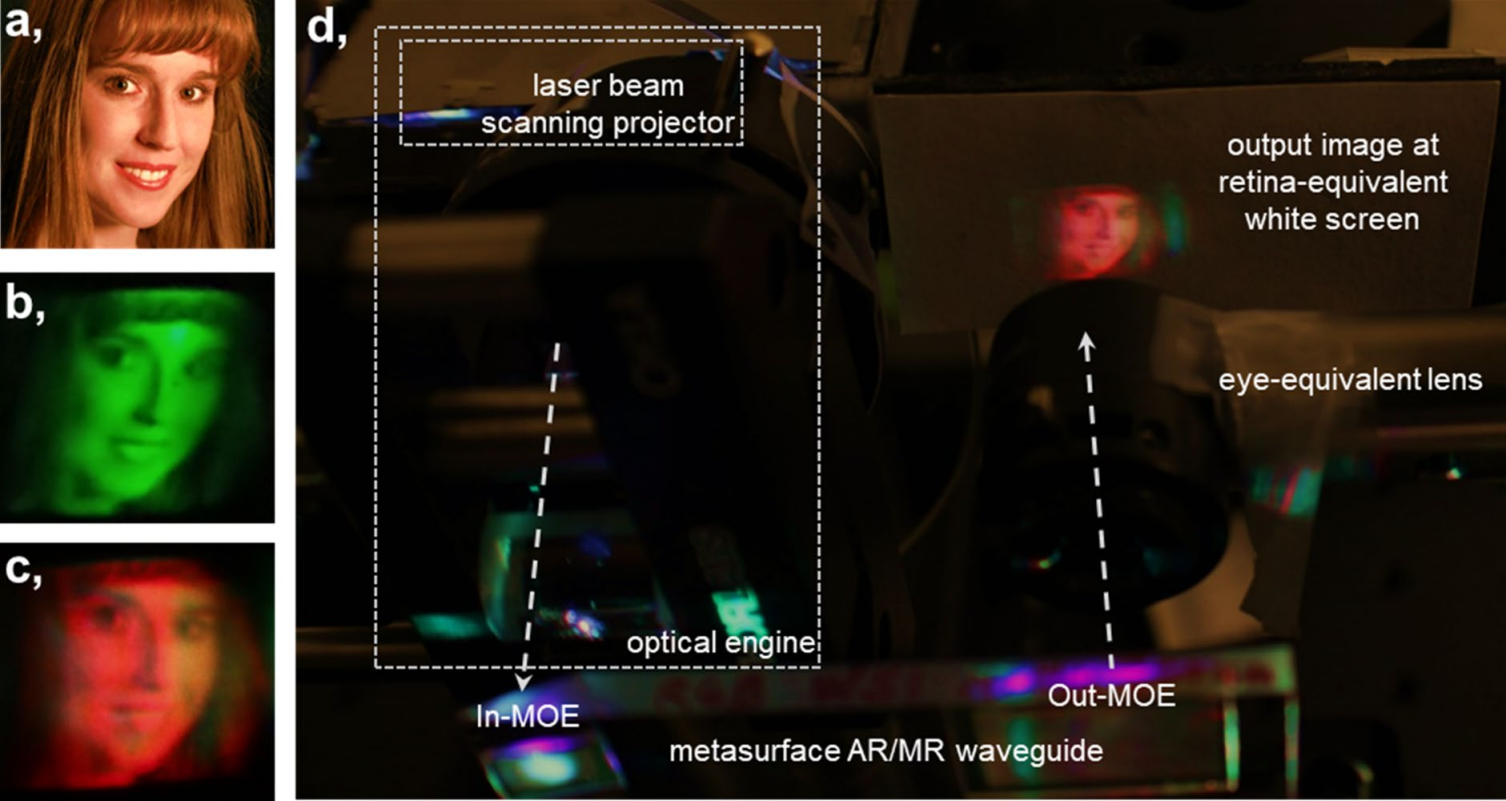
Experimental proof-of-principle observations of our metasurface waveguide display. (**a**) Original input image, from the International Committee for Display Metrology (ICDM) test pattern, sourced from VESA flat-panel display measurement (FPDM) standard. (**b**) Green mono-MOE smart glass output green image with only green laser input. (**c**) Color-MOE smart glass output image with red, green and blue laser input. (**d**) Optical engine and optical lens coupling interface—metasurface demonstration, with projected image at the retina-equivalent white screen location. Note that the camera photographs (**b**–**d**) and documents printouts are displayed in lower resolution than actual viewing in live-operation. The modulation transfer function (Fig. 6) is the more rigorous demonstration of the metasurface glass waveguide display performance.

**Figure 8:**
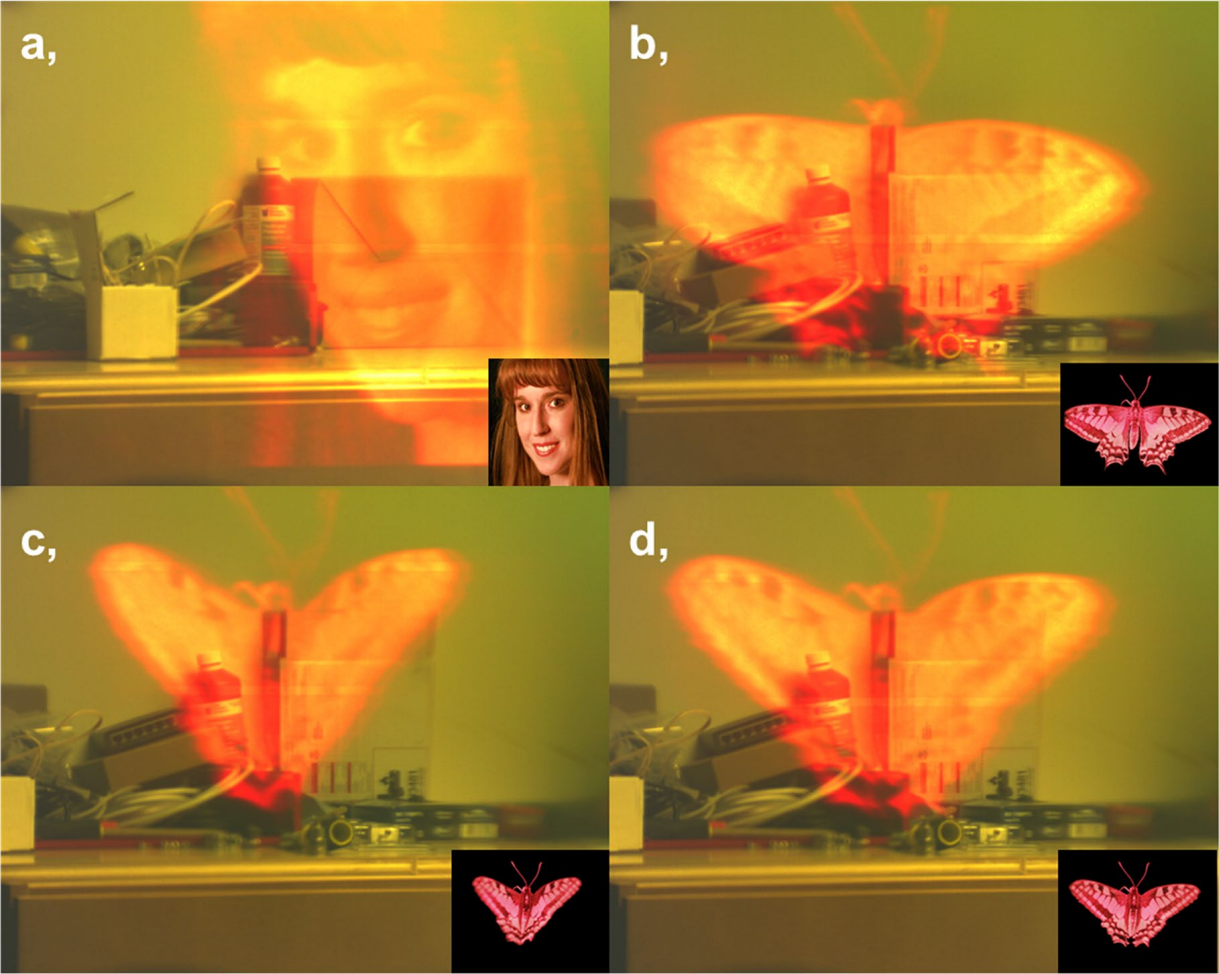
Display results from our metasurface waveguide display. (**a**–**d**) Inset shows the original images displayed. Observed images captured via a CMOS camera. The superimposed image of panel (**a**) is from the International Committee for Display Metrology (ICDM) test pattern, sourced from VESA flat-panel display measurement (FPDM) standards.

With the MTF determined and as proof-of-principle, we build up a test measurement system consisting of the optical engine, the MOEs (either the green mono-MOE or color-MOE prototypes), an eye-equivalent lens, and a retina-equivalent white screen as shown in Fig. 7. An example input image at 1080 × 720 pixels is illustrated in Fig. 7a. For the green mono-MOE, only the green laser in our laser beam scanning projector is turned on and therefore only the green channel of the original input image (Fig. 7a) is projected through the whole system on the retina-equivalent white screen. This is depicted in Fig. 7b, for the metasurface display demonstration. We note that the boundary edges of the green image are not captured, and this is due to the cylinder lens pair size in our metasurface demonstration. For the color-MOE, all lasers (red, green and blue) in the laser beam scanning projector are turned on and therefore all RGB channels of the original input image are projected through our metasurface waveguide demonstration. This is illustrated in Fig. 7c, as a proof-of-principle. The image is reddish as the red segments of the color-MOE currently have higher efficiency than the green and blue segments. This can be re-balanced by optimizing the RGB efficiency of our color-MOE. To show the capability of our MOE on real world application, Fig. 8 illustrates the augmented reality image captured on a CMOS camera. The image was observed on a red mono-MOE prototype with the Sony laser projector for the optical engine. We also note that main cause of the image degradation is from the mismatch between the optical engine and MOEs, introducing spherical and chromatic aberrations, astigmatism, field curvature and distortion. This formation of the multi-pixel displays across the input MOE, output MOE, waveguide, optical engine, and imaging sub-system, however, paves the platform of nanostructured metasurfaces towards potential AR displays.

Table 1 summarizes and compares the performance of our prototype with prototypical AR/MR waveguide displays^29,133–138^. These prior approaches use HOE-DOE and waveguides, typically consisting of in-coupling, intermediate, and out-coupling stages. They have, however, low output efficiencies (<< 1%) since their multiple diffraction elements and waveguides generate more undesired diffraction light, while our display architecture can be achieved with one waveguide and two MOEs. The prior DOEs also typically have smaller horizontal FoVs, below 40°. The prior multi-layer grating structures are also more challenging for scaling up the fabrication. The current limitation of our single-layer two MOE implementation is a smaller eyebox, resulting in cutting of the virtual image with eye movement from the center position. However, mechanical or optical methods for eyebox increase are currently widely researched and more advanced concepts on the metasurfaces including eye-tracking^139^, increasing view-points^140^ can potentially overcome this. We also note the prior implementations are single-focal or dual focal—with the user only observing the virtual object clearly when focusing their eyes to the plane. In other words, the user cannot see virtual objects at infinity if they are looking a close-by object. In contrast, our MOE architecture is by design focal-free and the image projected onto the retina, alleviating the vergence-accommodation conflict and enables the virtual object to be clearly seen whenever the user is focusing from near to infinity. Enabled by numerical design-optimization and foundry-based nanofabrication, we demonstrate experimental proof-of-principle operation of metasurface optical elements towards AR/MR waveguide displays. Our metasurfaces has enabled FoV greater than 40°, input–output efficiencies greater than 1% and is based on a focal-free implementation, in support of augmented display technologies.

**Table 1:** Performance comparison of our prototype design with prototypical AR/MR waveguide glasses.

	^129^	^130–133^	^134^	Our design
OE Display Type	DLP	LCoS	LCoS	LCoS/LBS
OE Display resolution	854 × 480	1280 × 720	1280 × 960	1280 × 720
OE display aspect ratio	16:9	16:9	4:3	16:9
Horizontal FOV	26.3°	30°	40°	≈ 40° to 55°
MTF contrast	≈ 35% for 854 × 480	NA	NA	> 25% for 1280 × 720
Efficiency	<< 1%	<< 1%	<< 1%	> 1%
Eyebox size	Large	Large	Large	Small
Number of waveguides	1	3	6	1
Number of MOE/HOEs	3	9	18	2
Waveguide thickness	≈ 1.3 mm	≈ 4 mm	≈ 2.8 mm	3 mm
Number of film layers	> 3	1	1	1
Focal planes	Single focal (infinity)	Single focal (infinity)	Dual focal (infinity and near)	Focal free (infinity to near)

## Methods

### Device fabrication

First a 350-nm Si-rich SiN_x_ layer is deposited on 500 μm thick fused silica wafers using low-pressure chemical vapor deposition (LPCVD, Tystar Titan II) with a gas mixture of SiH_2_Cl_2_ and NH_3_. The resulting silicon nitride layer was patterned via lithography at Broadcom Inc, by an optimized ASML PAS5500-1150 scanner capable of 90 nm resolution with 280 nm of positive resist and 40 nm of bottom anti-reflective coating or at UCSB Nanofab with a 248 nm DUV ASML 5500 stepper using a positive resist of UV210 and top anti-reflective coating DUV42P-6 with thicknesses of 230 nm and 60 nm, respectively. Subsequently the SiN_x_ layer is etched down at UCLA Nanolab using dry reactive ion etching via ULVAC NLD 570 fluorine etching machine using a photoresist etch mask. The etch parameters were 38 sccm Argon, 4 sccm oxygen, and 38 sccm CHF_3_, 700 W ICP power, and 100 W RIE power at a pressure of 3 mTorr. The Si_3_N_4_ etch was able to achieve a 3:1 aspect ratio with 150 nm feature sizes and a side wall angel of 86 degrees with a mask selectivity of 1.8:1. and diced. Each input–output MOE is then bonded to the glass waveguide for testing. Separate input–output MOEs are examined via SEM (Hitachi S4700) for sidewall and dimensional characterization.

## References

[1] Kress, B. C. Digital optical elements and technologies (EDO19): applications to AR/VR/MR. In *Proceedings of SPIE 11062, Digital Optical Technologies 2019*, Vol. 1106222 (2019).

[2] Bottani E, Vignali G (2019). Augmented reality technology in the manufacturing industry: A review of the last decade. IISE Trans..

[3] Dey A, Billinghurst M, Lindeman RW, Swan JE (2018). A systematic review of 10 years of augmented reality usability studies: 2005 to 2014. Front. Robot. AI.

[4] Kress, B. C. & Shin, M. Diffractive and holographic optics as optical combiners in head mounted displays. In *UbiComp '13 Adjunct: Proceedings of the 2013 ACM conference on Pervasive and ubiquitous computing adjunct publication*, 1479 (2013).

[5] Berryman DR (2012). Augmented reality: A review. Med. Ref. Serv. Q..

[6] Rolland J, Cakmakci O (2009). Head-worn displays: The future through new eyes. Opt. Photon. News.

[7] Orange-Kedem R, Nehme E, Weiss LE, Ferdman B, Alalouf O, Opatovski N, Shechtman Y (2021). 3D printable diffractive optical elements by liquid immersion. Nat. Commun..

[8] Goncharsky A, Goncharsky A, Melnik D, Durlevich S (2021). Nanooptical elements for visual verification. Sci. Rep..

[9] Yu Y, Sun C, Hsieh P, Huang Y, Song C, Yang T (2020). An edge-lit volume holographic optical element for an objective turret in a lensless digital holographic microscope. Sci. Rep..

[10] Banerji S, Cooke J, Sensale-Rodriguez B (2020). Impact of fabrication errors and refractive index on multilevel diffractive lens performance. Sci. Rep..

[11] Goncharsky A, Durlevich S (2020). DOE for the formation of the effect of switching between two images when an element is turned by 180 degrees. Sci. Rep..

[12] Cho SY, Ono M, Yoshida H, Ozaki M (2020). Bragg-Berry flat reflectors for transparent computer-generated holograms and waveguide holography with visible color playback capability. Sci. Rep..

[13] Wang H, Piestun R (2020). Azimuthal multiplexing 3D diffractive optics. Sci. Rep..

[14] Arns JA, Colburn WS, Barden SC (1998). Volume phase gratings and their potentials for astronomical applications. Proc. SPIE.

[15] Blanche BP, Gailly P, Habraken S, Lemaire P, Jamar C (2004). Volume phase holographic gratings: large size and high diffraction efficiency. Opt. Eng..

[16] Mukawa H, Akutsu K, Matsumura I, Nakano S, Yoshida T, Kuwahara M, Aiki K (2009). A full color eyewear display using planar waveguides with reflection volume holograms. J. Soc. Inf. Disp..

[17] Guo J, Tu Y, Yang L, Wang L, Wang B (2015). Design of a multiplexing grating for color holographic waveguide. Opt. Eng..

[18] Wu Z, Liu J, Wang Y (2013). A high-efficiency holographic waveguide display system with a prism in-coupler. J. Soc. Inf. Disp..

[19] Kämpfe T, Kley EB, Tünnermann A, Dannberg P (2007). Design and fabrication of stacked, computer generated holograms for multicolor image generation. Appl. Opt..

[20] Gupta MC, Peng ST (1993). Diffraction characteristics of surface-relief gratings. Appl. Opt..

[21] Kwan C, Taylor GW (1998). Optimization of the parallelogrammic grating diffraction efficiency for normally incident waves. Appl. Opt..

[22] Moharam MG, Gaylord TK (1982). Diffraction analysis of dielectric surface-relief gratings. J. Opt. Soc. Am. A.

[23] Preist TW, Harris JB, Wanstall NP, Sambles JR (1997). Optical response of blazed and overhanging gratings using oblique Chandezon transformations. J. Mod. Opt..

[24] Yokomori K (1984). Dielectric surface-relief gratings with high diffraction efficiencies. Appl. Opt..

[25] Tishchenko AV (2005). Phenomenological representation of deep and high contrast lamellar gratings by means of the modal method. Opt. Quantum Electron..

[26] Miller JM, de Beaucoudrey N, Chavel P, Turunen J, Cambril E (1997). Design and fabrication of binary slanted surface-relief gratings for a planar optical interconnection. Appl. Opt..

[27] Maikisch JS, Gaylord TK (2007). Optimum parallel-face slanted surface-relief gratings. Appl. Opt..

[28] Levola T, Laakkonen P (2007). Replicated slanted gratings with a high refractive index material for in and outcoupling of light. Opt. Express.

[29] Kress BC, Chatterjee I (2021). Waveguide combiners for mixed reality headsets: a nanophotonics design perspective. Nanophotonics.

[30] Richter, F. The Diverse Potential of VR & AR Applications. www.statista.com/chart/4602/virtual-and-augmented-reality-software-revenue (2016).

[31] Virtual & Augmented Reality: The Next Big Computing Platform? www.goldmansachs.com/insights/pages/virtual-and-augmented-reality-report.html (2016).

[32] Hall, S. & Takahashi, R. Augmented and virtual reality: The promise and peril of immersive technologies. *McKinsey Insight Article* (2017).

[33] Cakmakci O, Rolland J (2006). “Head-worn displays: A review”, Display Technology. J. Disp. Technol..

[34] Bharadwaj SR, Candy TR (2009). Accommodative and vergence responses to conflicting blur and disparity stimuli during development. J. Vis..

[35] Hoffman DM, Girshick AR, Akeley K, Banks MS (2008). Vergence-accommodation conflicts hinder visual performance and cause visual fatigue. J. Vis..

[36] Lambooij M, IJsselsteijn W, Fortuin M, Heynderickx I (2009). Visual discomfort and visual fatigue of stereoscopic displays: A review. J. Imaging Sci. Technol..

[37] Reichelt, S., Haussler, R., Futterer, G. & Leister, N. Depth cues in human visual perception and their realization in 3D displays. In *Proceedings of SPIE 7690, Three-Dimensional Imaging, Visualization, and Display 2010 and Display Technologies and Applications for Defense, Security, and Avionics IV*, Vol. 7690, 76900B (2010).

[38] Lin T, Zhan T, Zou J, Fan F, Wu S (2020). Maxwellian near-eye display with an expanded eyebox. Opt. Express.

[39] Chang C, Cui W, Park J, Gao L (2019). Computational holographic Maxwellian near-eye display with an expanded eyebox. Sci. Rep..

[40] Lee JS, Kim YK, Lee MY, Won YH (2019). Enhanced see-through near-eye display using time-division multiplexing of a Maxwellian-view and holographic display. Opt. Express.

[41] Takaki Y, Fujimoto N (2018). Flexible retinal image formation by holographic Maxwellian-view display. Opt. Express.

[42] Fan J, Cheng Y, He B (2021). High-efficiency ultrathin terahertz geometric metasurface for full-space wavefront manipulation at two frequencies. J. Phys. D Appl. Phys..

[43] Wei Q, Huang L, Zentgraf T, Wang Y (2020). Optical wavefront shaping based on functional metasurfaces. Nanophotonics.

[44] Wang Z, Li T, Soman A, Mao D, Kananen T, Gu T (2019). On-chip wavefront shaping with dielectric metasurface. Nat. Commun..

[45] Lee G, Hong J, Hwang S, Moon S, Kang H, Jeon S, Kim H, Jeong J, Lee B (2018). Metasurface eyepiece for augmented reality. Nat. Commun..

[46] Kildishev AV, Boltasseva A, Shalaev VM (2013). Planar photonics with metasurfaces. Science.

[47] Yu N, Capasso F (2014). Flat optics with designer metasurfaces. Nature Mater..

[48] Genevet P, Capasso F, Aieta F, Khorasaninejad M, Devlin R (2017). Recent advances in planar optics: From plasmonic to dielectric metasurfaces. Optica.

[49] Falcone F, Lopetegi T, Laso MAG, Baena JD, Bonache J, Beruete M, Marqués R, Martín F, Sorolla M (2004). Babinet principle applied to the design of metasurfaces and metamaterials. Phys. Rev. Lett..

[50] Hsiao HH, Chu CH, Tsai DP (2017). Fundamentals and applications of metasurfaces. Small Methods.

[51] Solntsev AS, Agarwal GS, Kivshar YS (2021). Metasurfaces for quantum photonics. Nat. Photonics.

[52] Zhou J, Liu S, Qian H, Li Y, Luo H, Wen S, Zhou Z, Guo G, Shi B, Liu Z (2020). Metasurface enabled quantum edge detection. Sci. Adv..

[53] Bekenstein R, Pikovski I, Pichler H, Shahmoon E, Yelin SF, Lukin MD (2020). Quantum metasurfaces with atom arrays. Nat. Phys..

[54] Georgi P, Massaro M, Luo K, Sain B, Montaut N, Herrmann H, Weiss T, Li G, Silberhorn C, Zentgraf T (2019). Metasurface interferometry toward quantum sensors. Light Sci. Appl..

[55] Altuzarra C, Lyons A, Yuan G, Simpson C, Roger T, Ben-Benjamin JS, Faccio D (2019). Imaging of polarization-sensitive metasurfaces with quantum entanglement. Phys. Rev. A.

[56] Ebbesen TW, Lezec HJ, Ghaemi H, Thio T, Wolff P (1998). Extraordinary optical transmission through sub-wavelength hole arrays. Nature.

[57] Yin L, Vlasko-Vlasov VK, Pearson J, Hiller JM, Hua J, Welp U, Brown DE, Kimball CW (2005). Subwavelength focusing and guiding of surface plasmons. Nano Lett..

[58] Liu Z, Steele JM, Srituravanich W, Pikus Y, Sun C, Zhang X (2005). Focusing surface plasmons with a plasmonic lens. Nano Lett..

[59] Huang FM, Zheludev N, Chen Y, Javier Garcia de Abajo F (2007). Focusing of light by a nanohole array. Appl. Phys. Lett..

[60] Sun Z, Kim HK (2004). Refractive transmission of light and beam shaping with metallic nano-optic lenses. Appl. Phys. Lett..

[61] Shi H, Wang C, Du C, Luo X, Dong X, Gao H (2005). Beam manipulating by metallic nano-slits with variant widths. Opt. Express.

[62] Verslegers L, Catrysse PB, Yu Z, White JS, Barnard ES, Brongersma ML, Fan S (2009). Planar lenses based on nanoscale slit arrays in a metallic film. Nano Lett..

[63] Yu N, Genevet P, Kats MA, Aieta F, Tetienne J, Capasso F, Gaburro Z (2011). Light propagation with phase discontinuities: Generalized laws of reflection and refraction. Science.

[64] Aieta F, Genevet P, Yu N, Kats MA, Gaburro Z, Capasso F (2012). Out-of-plane reflection and refraction of light by anisotropic optical antenna metasurfaces with phase discontinuities. Nano Lett..

[65] Aieta F, Genevet P, Kats MA, Yu N, Blanchard R, Gaburro Z, Capasso F (2012). Aberration-free ultrathin flat lenses and axicons at telecom wavelengths based on plasmonic metasurfaces. Nano Lett..

[66] Memarzadeh B, Mosallaei H (2011). Array of planar plasmonic scatterers functioning as light concentrator. Opt. Lett..

[67] Ni X, Ishii S, Kildishev AV, Shalaev VM (2013). Ultra-thin, planar, Babinet-inverted plasmonic metalenses. Light Sci. Appl..

[68] Nikolov DK, Bauer A, Cheng F, Kato H, Vamivakas AN, Rolland JP (2021). Metaform optics: Bridging nanophotonics and freeform optics. Sci. Adv..

[69] Bayati E, Wolfram A, Colburn S, Huang L, Majumdar A (2021). Design of achromatic augmented reality visors based on composite metasurfaces. Appl. Opt..

[70] Nikolov DK, Cheng F, Ding L, Bauer A, Vamivakas AN, Rolland JP (2019). See-through reflective metasurface diffraction grating. Opt. Mater. Express.

[71] Brongersma ML (2021). The road to atomically thin metasurface optics. Nanophotonics.

[72] Zou H, Li P, Peng P (2020). An ultra-thin acoustic metasurface with multiply resonant units. Phys. Lett. A.

[73] Guo X, Ding Y, Duan Y, Ni X (2019). Nonreciprocal metasurface with space–time phase modulation. Light Sci. Appl..

[74] Kang M, Feng T, Wang H-T, Li J (2012). Wave front engineering from an array of thin aperture antennas. Opt. Express.

[75] Chen X, Huang L, Mühlenbernd H, Li G, Bai B, Tan Q, Jin G, Qiu C, Zhang S, Zentgraf T (2012). Dual-polarity plasmonic metalens for visible light. Nat. Commun..

[76] Sun S, Yang K, Wang C, Juan T, Chen WT, Liao CY, He Q, Xiao S, Kung W, Guo G, Zhou L, Tsai DP (2012). High-efficiency broadband anomalous reflection by gradient meta-surfaces. Nano Lett..

[77] Sun S, He Q, Xiao S, Xu Q, Li X, Zhou L (2012). Gradient-index meta-surfaces as a bridge linking propagating waves and surface waves. Nat. Mater..

[78] Pors A, Nielsen MG, Eriksen RL, Bozhevolnyi SI (2013). Broadband focusing flat mirrors based on plasmonic gradient metasurfaces. Nano Lett..

[79] Zhang S, Kim M, Aieta F, She A, Mansuripur T, Gabay I, Khorasaninejad M, Rousso D, Wang X, Troccoli M, Yu N, Capasso F (2016). High efficiency near diffraction-limited mid-infrared flat lenses based on metasurface reflect arrays. Opt. Express.

[80] Monticone F, Estakhri NM, Alù A (2013). Full control of nanoscale optical transmission with a composite metascreen. Phys. Rev. Lett..

[81] Jahani S, Jacob Z (2016). All-dielectric metamaterials. Nat. Nanotechnol..

[82] Kuznetsov AI, Miroshnichenko AE, Brongersma ML, Kivshar YS, Luk’yanchuk B (2016). Optically resonant dielectric nanostructures. Science.

[83] Flanders DC (1983). Submicrometer periodicity gratings as artificial anisotropic dielectrics. Appl. Phys. Lett..

[84] Hasman E, Kleiner V, Biener G, Niv A (2003). Polarization dependent focusing lens by use of quantized Pancharatnam—Berry phase diffractive optics. Appl. Phys. Lett..

[85] Levy U, Kim H-C, Tsai C-H, Fainman Y (2005). Near-infrared demonstration of computer-generated holograms implemented by using subwavelength gratings with space-variant orientation. Opt. Lett..

[86] Lin D, Fan P, Hasman E, Brongersma ML (2014). Dielectric gradient metasurface optical elements. Science.

[87] Khorasaninejad M, Chen WT, Devlin RC, Oh J, Zhu AY, Capasso F (2016). Metalenses at visible wavelengths: Diffraction-limited focusing and subwavelength resolution imaging. Science.

[88] Luo W, Xiao S, He Q, Sun S, Zhou L (2015). Photonic spin Hall effect with nearly 100% efficiency. Adv. Opt. Mater..

[89] Zheng G, Mühlenbernd H, Kenney M, Li G, Zentgraf T, Zhang S (2015). Metasurface holograms reaching 80% efficiency. Nat. Nanotechnol..

[90] Kress BC, Meyrueis P (2009). Applied Digital Optics: From Micro-optics to Nanophotonics.

[91] Lu F, Sedgwick FG, Karagodsky V, Chase C, Chang-Hasnain CJ (2010). Planar high-numerical-aperture low-loss focusing reflectors and lenses using subwavelength high contrast gratings. Opt. Express.

[92] Fattal D, Li J, Peng Z, Fiorentino M, Beausoleil RG (2010). Flat dielectric grating reflectors with focusing abilities. Nat. Photonics.

[93] Arbabi A, Horie Y, Ball AJ, Bagheri M, Faraon A (2015). Subwavelength-thick lenses with high numerical apertures and large efficiency based on high-contrast transmit arrays. Nat. Commun..

[94] West PR, Stewart JL, Kildishev AV, Shalaev VM, Shkunov VV, Strohkendl F, Zakharenkov YA, Dodds RK, Byren R (2014). All-dielectric subwavelength metasurface focusing lens. Opt. Express.

[95] Shalaginov MY, An S, Zhang Y, Yang F, Su P, Liberman V, Chou JB, Roberts CM, Kang M, Rios C, Du Q, Fowler C, Agarwal A, Richardson KA, Rivero-Baleine C, Zhang H, Hu J, Gu T (2021). Reconfigurable all-dielectric metalens with diffraction-limited performance. Nat. Commun..

[96] Balli F, Sultan M, Lami SK, Hastings JT (2020). A hybrid achromatic metalens. Nat. Commun..

[97] Presutti F, Monticone F (2020). Focusing on bandwidth: Achromatic metalens limits. Optica.

[98] Lin RJ, Su V, Wang S, Chen MK, Chung TL, Chen YH, Kuo HY, Chen J, Chen J, Huang Y, Wang J, Chu CH, Wu PC, Li T, Wang Z, Zhu S, Tsai DP (2019). Achromatic metalens array for full-colour light-field imaging. Nat. Nanotechnol..

[99] Zhan A, Colburn S, Trivedi R, Fryett TK, Dodson CM, Majumdar A (2016). Low-contrast dielectric metasurface optics. ACS Photonics.

[100] Estakhri NM, Neder V, Knight MW, Polman A, Alù A (2017). Visible light, wide-angle graded metasurface for back reflection. ACS Photonics.

[101] Khorasaninejad M, Zhu AY, Roques-Carmes C, Chen WT, Oh J, Mishra I, Devlin RC, Capasso F (2016). Polarization-insensitive metalenses at visible wavelengths. Nano Lett..

[102] Devlin RC, Khorasaninejad M, Chen WT, Oh J, Capasso F (2016). Broadband high-efficiency dielectric metasurfaces for the visible spectrum. Proc. Natl. Acad. Sci..

[103] Khorasaninejad M, Chen WT, Zhu AY, Oh J, Devlin RC, Roques-Carmes C, Mishra I, Capasso F (2017). Visible wavelength planar metalenses based on titanium dioxide. IEEE J. Sel. Top. Quantum Electron..

[104] Vo S, Fattal D, Sorin WV, Peng Z, Tran T, Fiorentino M, Beausoleil RG (2014). Sub-wavelength grating lenses with a twist. IEEE Photonics Technol. Lett..

[105] Arbabi A, Arbabi E, Kamali SM, Horie Y, Han S, Faraon A (2016). Miniature optical planar camera based on a wide-angle metasurface doublet corrected for monochromatic aberrations. Nat. Commun..

[106] Groever B, Chen WT, Capasso F (2017). Meta-lens doublet in the visible region. Nano Lett..

[107] Chen WT, Zhu AY, Khorasaninejad M, Shi Z, Sanjeev V, Capasso F (2017). Immersion meta-lenses at visible wavelengths for nanoscale imaging. Nano Lett..

[108] Clausnitzer T, Kämpfe T, Kley EB, Tünnermann A, Peschel U, Tishchenko AV, Parriaux O (2005). An intelligible explanation of highly-efficient diffraction in deep dielectric rectangular transmission gratings. Opt. Express.

[109] Zheng J, Zhou C, Feng J, Wang B (2008). Polarizing beam splitter of deep-etched triangular-groove fused-silica gratings. Opt. Lett..

[110] Braig CC, Fritzsch L, Kasebier T, Kley E-B, Laubis C, Liu Y, Scholze F, Tunnermann A (2012). An EUV beamsplitter based on conical grazing incidence diffraction. Opt. Express.

[111] Wan C, Gaylord TK, Bakir MS (2016). Rigorous coupled-wave analysis equivalent-index-slab method for analyzing 3D angular misalignment in interlayer grating couplers. Appl. Opt..

[112] Moharam MG, Grann EB, Pommet DA (1995). Formulation for stable and efficient implementation of the rigorous coupled-wave analysis of binary gratings. J. Opt. Soc. Am. A.

[113] Moharam MG, Pommet DA, Grann EB, Gaylord TK (1995). Stable implementation of the rigorous coupled wave analysis for surface-relief dielectric gratings: enhanced transmittance matrix approach. J. Opt. Soc. Am. A.

[114] Moharam MG, Gaylord TK (1981). Rigorous coupled-wave analysis of planar-grating diffraction. J. Opt. Soc. Am..

[115] Wan C, Gaylord TK, Bakir MS (2016). Grating design for interlayer optical interconnection of in-plane waveguides. Appl. Opt..

[116] Allured R, McEntaffer RT (2013). Analytical alignment tolerances for off-plane reflection grating spectroscopy. Exp. Astron..

[117] Eisen L, Meyklyar M, Golub M, Friesem AA, Gurwich I, Weiss V (2006). Planar configuration for image projection. Appl. Opt..

[118] Cameron A (2009). The application of holographic optical waveguide technology to Q-sight family of helmet mounted displays. Proc. SPIE.

[119] Kogelnik H (1969). Coupled-wave theory of thick hologram gratings. Bell Syst. Tech. J..

[120] Sheng P, Stepleman RS, Sanda PN (1982). Exact eigenfunctions for square-wave gratings—Application to diffraction and surface-plasmon calculations. Phys. Rev. B.

[121] Oskooi A, Roundy D, Ibanescu M, Bermel P, Joannopoulos JD, Johnson SG (2010). MEEP: A flexible free-software package for electromagnetic simulations by the FDTD method. Computer Phys. Commun..

[122] Lee, S., Abràmoff, M. D. & Reinhardt, J. M. Retinal image mosaicing using the radial distortion correction model. In *International Society for Optics and Photonics* 691435 (2008).

[123] Li J, Su J, Zeng X (2019). A solution method for image distortion correction model based on bilinear interpolation. Comput. Opt..

[124] Chen Z, Sang X, Li H, Wang Y, Zhao L (2019). Ultra-lightweight and wide field of view augmented reality virtual retina display based on optical fiber projector and volume holographic lens. Chin. Opt. Lett..

[125] Martinez, C., Krotov, V., Fowler, D. & Haeberlé, O. Lens-free near-eye intraocular projection display, concept and first evaluation. In *Computational Optical Sensing and Imaging, CW1C*, Vol. 5 (2016).

[126] Matthies DJ, Haescher M, Alm R, Urban B (2015). Properties of a peripheral head-mounted display (phmd). Int. Conf. Hum. Comput. Interact..

[127] Boreman GD (2001). Modulation Transfer Function in Optical and Electro-optical Systems.

[128] Barten PG (1992). Physical model for the contrast sensitivity of the human eye. Int. Soc. Opt. Photonics.

[129] Barten PG (1999). Contrast Sensitivity of the Human Eye and Its Effects on Image Quality.

[130] Barten PG (2003). Formula for the contrast sensitivity of the human eye. Proc. SPIE.

[131] Kress BC (2019). Digital optical elements and technologies (EDO19): Applications to AR/VR/MR. Int. Soc. Opt. Photonics.

[132] Guo Y, Yao G, Lei B, Tan J (2008). Monte Carlo model for studying the effects of melanin concentrations on retina light absorption. J. Opt. Soc. Am. A.

[133] Zhang Y, Fang F (2019). Development of planar diffractive waveguides in optical see-through head-mounted displays. Precis. Eng..

[134] Kress, B. C. Optical waveguide combiners for AR headsets: Features and limitations. In *Proceedings of SPIE 11062, Digital Optical Technologies 2019*, Vol. 11062, 110620J (2019).

[135] Kress BC, Cummings WJ (2017). 11–1: invited paper: Towards the ultimate mixed reality experience: HoloLens display architecture choices. SID Symp. Dig. Tech. Pap..

[136] Kress BC, Cummings WJ (2017). Optical architecture of HoloLens mixed reality headset. Proc. SPIE.

[137] Microsoft Hololens, Mixed Reality Technology for Business.

[138] Magic Leap, Augmented reality platform for Enterprise.

[139] Kim J, Jeong Y, Stengel M, Akşit K, Albert R, Boudaoud B, Greer T, Kim J, Lopes W, Majercik Z, Shirley P, Spjut J, McGuire M, Luebke D (2019). Foveated AR: Dynamically-foveated augmented reality display. ACM Trans. Graph..

[140] Jo Y, Yoo C, Bang K, Lee B, Lee B (2021). Eye-box extended retinal projection type near-eye display with multiple independent viewpoints. Appl. Opt..

